# A Case of Epilepsy Cured (Apparently) by the Correction of an Error of Refraction

**Published:** 1888-06

**Authors:** J. Elliott Colburn

**Affiliations:** Chicago, Ill.; Professor of Ophthalmology and Otology in Chicago Polyclinic; Ophthalmic Surgeon to Cook County Hospital; Assistant Surgeon Illinois State Charitable Eye and Ear Infirmary, etc.


### A CASE OF EPILEPSY CURED (APPARENTLY) BY THE CORRECTION OF AN ERROR OF REFRACTION.*

BY J. ELLIOTT COLBURN, M. D.,

Professor of Ophthalmology and Otology in Chicago Polyclinic; Ophthalmic Surgeon to Cook County Hospital; Assistant Surgeon Illinois State Charitable Eye and Ear Infirmary, etc.

The following case, to which I wish to call your attention, first came under my observation in June, 1882:

Mr. T., æt. 24 years, 5 feet 9 inches in height, weight 160 pounds, well proportioned, features regular, except that eyes are deeply set and appear small; occuation student.

He consulted me for mental confusion and inability to read or study without flushing of face and pulsating of superficial arteries, with sensation of pounding in back of head and neck, with marked sensations of vertigo which would disappear when eyes were closed. At times, following prolonged effort, the confusion of ideas was painful and was accompanied by visions which he recognized as such, at least a part of the time. He had at this time some slight disturbance of digestion and constipation, due apparently to sedentary life. He admitted masturbation in early boyhood, but not since; had had frequent seminal emissions, more marked during the past two years and always following hard study. I gave him such treatment as his case seemed to demand, with the result of benefiting his digestion; otherwise the case

*Read before the Chicago Medical Society, January 16th, 1888.

remained about the same. About this time, for some conduct not in accord with the rules of the institution, he was brought before the faculty. The President, finding that he had consulted me, came to find out whether the gentleman was really ill, or whether there was good reason for his strange actions and low standing in the school. From him I learned that the patient was constantly manifesting strange mental phenomena, by them attributed to viciousness and low mental and moral character. At times he was extremely dull and morose, and again almost brilliant and exemplary.

I then inquired more carefully into the case from himself and others, and diagnosed a condition bordering on *petit mal* with impulses. I also found that he was unusually bright as a critic of orations and sermons, while it was with difficulty that he was able to grasp the matter of a printed page. He was advised to leave school for some occupation less sedentary and requiring less mental work. This he did. At the end of the summer vacation he returned apparently perfectly well.

About this time I left that field of work and lost track of my patient. From a student in the institution at that time I learned that for some weeks following his reëntrance he did good school work, but resumed his old habits and, after a stormy time with the faculty, left the school. After some difficulty he was admitted to the ministry, for which he had been preparing. About two years subsequent to my first acquaintance with him, during a sermon, he was attacked with a marked epileptic seizure, and from that time on he was subject to attacks more or less severe, occurring in the frequency of from one a day to one in two or three weeks.

Restraint was considered advisable, and he was confined in an asylum at intervals for about two years. During one of his more prolonged intervals of immunity from fits, he escaped from the asylum and went to a neighboring State, where he endeavored to resume his work, but the return of the malady again unfitted him for ministerial labor and he resorted to canvassing. The study necessary for success in this field soon increased his trouble.

About this time he recalled my opinion and prognosis of his case, and finding my address called upon me for advice. He came to me in August, 1886. I found him at my office in a truly pitiable plight. He had been a number of hours on the cars and had had numerous seizures more or less severe; he was hungry, bewildered and incoherent, with marked delusions. I hastened to put him in better condition, and on the following day elicited some of the facts given above.

His trouble continued in spite of sedatives, and I referred him to Dr. C. D. Wescott for examination and advice. Dr. Wescott gives me the following notes of his case:

"Mr. T. first called upon me October 23, 1886. I pronounced him an epileptic as he entered my office, from his appearance alone, the congested countenance and excessively mobile pupil. He described very well seizures of *petit mal* occurring every two or three weeks, with or without unconsciousness, frequently followed by uncontrollable impulses and hallucinations. His judgment was evidently impaired, and he presented every appearance of one suffering from the effect of that disease. Among the prominent symptoms was his inability to read without the greatest fatigue and general nervous irritation. I put him on small doses of the mixture of bromides and gave him careful directions as to diet. The bromides he took only a short time. I gave him a stomach tonic and laxative. He appeared to improve for a short time and then remained in about the same condition, always being irritated by any attempt at mental effort.

"The only attack which I know of his having, following Dr. Colburn's treatment, was about January 25, 1887. He came into my office feeling unusually well, jovial and lively. A little incoördination was noticeable in his gait and, in spite of my acquaintance with the precursor of epileptic mania, I was inclined to suspect alcohol. After assuring myself that he had had no liquor, I prescribed saline cathartics and sent him home. In the evening I was sent for and found him in a condition of mild mania, with delusions and hallucinations and a fear of impending evil. The attack was easily controlled with chloral and bromides and he was soon about again, but for some days was dull and depressed and incapable of mental or physical exertion. Since the above indicated attack I have seen him but seldom, and not at all of late, but when I talked with him last his mind seemed stronger, his articulation was more natural, and his countenance wore a more intelligent and natural expression."—C. D. WESCOTT.

On referring to some private notes made in 1882, I found that I had at that time examined his eyes and obtained .50 D. manifest and 2 D. latent hypermetropia. I at once acted on the suggestion of Dr. George T. Stevens, of New York, who had kindly admitted me to his private cases. I atropinized my patient, verifying my former examination. Also found insufficiency of the external recti equal to 8°, which was treated by exercise with the prisms, soon obtaining a more normal balance of the recti muscles, and prescribed a + 2 D. for constant use. He was directed to discontinue the treatment instituted by Dr. Wescott. From this time on there was a marked improvement of all his symptoms, mental and physical. During January, 1887, he had a marked attack, as mentioned by Dr. Wescott, but at that time there was also quite an increase of temperature and disturbance of stomach and bowels, showing that the possible exciting cause of this attack was some malarial disturbance.

For about one year he has had nothing resembling his former trouble. His mind has become active and his personal appearance changed to that of a man in perfect health, and he is now a perfectly well man, taking long trips through the country, the trusted employé of one of our large publishing houses. For about a year he has used his glasses for near work only.

In June, 1887, my patient brought his mother to the city for advice. I referred her to Dr. Lyman, and from him have the following notes:

"The case is one of neurasthenia with tendency to marked epileptic convulsions with verti go, tottering and unsteadiness of gait."

Dr. Lyman referred the case to me for an examination of the eyes. I found on examination that there was hypermetropia of 2 D., 1 D. ast. and presbyopia of 2 D., all of which I corrected. Her daughter, who accompanied her, was emmetropic with no nervous tendencies, and resembled her father, while Mr. T. very strongly resembled his mother. Since the treatment instituted by Dr. Lyman, iron, quinine, strychnia, and the constant use of glasses for distance, the mother has improved and is now quite strong and well.

Mr. T. has a brother now confined in an asylum for some form of insanity. I have written for the history of this case. I learned from the mother that the brother of my patient was subject to sick headaches, school headaches, etc., and that he was an erratic child, gradually increasing in peculiar tendencies until marked mental disturbance was developed.

To me the point of special significance is: *The hereditary hypermetropic* error of refraction; in the mother, apparently causing nervous prostration; in the son, all of the progressive symptoms of cerebral disturbance up to mania; the apparently direct relation, in case of Mr. T., between the amount of work done and the progressive congestion of face and brain; that any return to the use of the eyes would precipitate severe nervous explosions, and that the attacks were seldom caused by other irritation; that the correction of the hypermetropia was followed by a great improvement till, within four months from the time of the adjustment, the glasses had so far relieved his trouble that he had no nervous explosions to date. Headaches, indigestion, constipation and seminal emissions are perfectly relieved, and the patient is able to do a large amount of work and read without trouble. In a letter just received from Mr. T. he says:

"Although I have always had a desire to be a student of general knowledge, yet, whenever I attended school, I was considered lazy and entirely out of my place, and until the latter part of my school days, I was made sport of, both by my associates and instructors, because they could see no excuse for me, as I seemed to be in perfect health.

"Later on my friends noticed that my face was flushed at times and my general dullness of expression. Many accused me of taking opium.

"In recitation I always stood the lowest in my class, but in examinations my standing was a great deal better, which, of course, my instructors were at a loss to know how to account for. I do not think that the cause of my poor recitations was laziness, but it was owing to the difficulty which I had in reading. Even aside from the dry reading of text-books I was never able to read more than a few minutes at a time without suffering pain from my eyes to the back of my head, and, in fact, my whole head seemed to be very much irritated, which made me flighty at times. I would have impulses to jump and scream. These symptoms of disorder were more severe if I did any reading in the evening.

"While sitting alone or lying in bed I would see flashes of light, imagine things, and in my mind would go on a journey and perform dare-devil feats. At times I would be conscious of being in my room during such trains of thought, and at others, not. Sleep was an impossibility till 3 or 4 o'clock in the morning.

"This trouble grew upon me until I was forced to leave school. Then I spent my time in going from place to place, preaching as best I could, but doing very little reading, and I thought I was getting better.

"About two years and a half ago I was called to attend a funeral. For a few weeks previous to this call I had not been feeling as well as usual. At first I refused to go, but I was finally persuaded to go upon a promise that little would be required of me. I had read but a few sentences from the book which is used on such occasions, when I saw a quick succession of dark waves pass before my eyes; I lost control of my muscles, and the next I knew I was lying on a bed in the next room. Dr. —, who was standing by me at the time, said that all he noticed until my muscles gave way was that my face flushed a little. However, I was soon able to finish the service and go home. These spells became more frequent and severe till finally I was put in an asylum.

"So far as I know, I never had a convulsion, and was not always wholly unconscious, but always lost control of my muscles.

"They never came on unless I overdid in some way, either physically or mentally, or under some excitement.

"The asylum gave me temporary relief, but after I had been out a short time the trouble returned; and until I had put myself under your treatment for a few months, I did not think there was any help for me. Since then I have gained in health, and am now able to do a good day's work without any symptoms of the old trouble."

*126 State Street.*

